# AI revolutions in biology: The joys and perils of AlphaFold

**DOI:** 10.15252/embr.202154046

**Published:** 2021-10-20

**Authors:** Anastassis Perrakis, Titia K Sixma

**Affiliations:** ^1^ Oncode Institute and Division of Biochemistry The Netherlands Cancer Institute Amsterdam The Netherlands

**Keywords:** Methods & Resources, Structural Biology

## Abstract

AlphaFold is the most ground‐breaking application of AI in science so far; it will revolutionize structural biology, but caution is warranted.

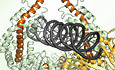

Last December, the artificial intelligence (AI) AlphaFold achieved what had been considered impossible: near‐perfect protein fold predictions. The shockwaves reverberated well beyond the scientific community: one of the grand challenges in biology, how to fold a protein, appeared to have been solved finally—something that most of us did not expect to happen in our lifetimes. Notwithstanding all the justified excitement about AlphaFold, this achievement does not mean though that AI will make experimental structural biology or its practitioners and tools redundant. Structural biology will remain essential for understanding how proteins work and how they dynamically interact with each other.

The publications of the AlphaFold method (Jumper *et al*, 2021) by the Google DeepMind team, and the analogous RoseTTAfold approach by the Baker laboratory at the University of Washington, Seattle, USA (Baek *et al*, 2021), a few months later, explain the background behind Deep Learning (DL) algorithms that made this scientific milestone possible. Detailed descriptions of the algorithm, the “trained” code for making new predictions, and even the training code to “learn” how to predict new structures, are all available to the public: protein prediction for the masses.

The DeepMind team, collaborating with the European Bioinformatics Institute (EBI), then applied AlphaFold to whole genomes, including human, mouse, *Saccharomyces,* and *E*. *coli* (Tunyasuvunakool *et al*, 2021). The resulting structure predictions are accessible via an elegant and informative Web interface (https://alphafold.ebi.ac.uk/) that gives structural biologists a “treasure trove” of new information. Here, we try to address the scope and implications of this watershed moment in structural biology.

## The protein folding problem

To better understand the enormous advance that AlphaFold and RoseTTAfold have achieved, it is worthwhile to look at the problem. Protein folding involves rearranging a linear sequence of amino acids in space to a physiologically preferred low‐energy state. Predicting the correct structure from the amino acid sequence alone was deemed an intractable problem, as the degrees of freedom in the peptide bonds create an astronomically high number of possible conformations: Sequential sampling would take longer than the age of the universe, even for a small protein domain (Levinthal, 1969).

… one of the grand challenges in biology, how to fold a protein, appeared to have been solved finally—something that most of us did not expect to happen in our lifetimes

Computational predictions were developed to circumvent the sequential sampling problem. They have been steadily improving over the past 40 years, using comparative sequence analysis to find homologies with the ever‐increasing number of experimentally determined protein structures by methods such as X‐ray crystallography, nuclear magnetic resonance spectroscopy (NMR), and cryogenic electron microscopy (cryo‐EM) that are publicly available from the Protein Data Bank (PDB) (Berman *et al*, 2003; Burley *et al*, 2019). In 1994, the community started a bi‐annual assessment of computational prediction methods to evaluate their performance by applying these to recently resolved protein structures before they became publicly available (Moult *et al*, 1995). Those four decades saw steady progress in performance, but the first implementation of AlphaFold three years ago was already a revolutionary advance by applying AI to the problem. In 2020, the near‐perfect performance of its re‐designed reincarnation created a seismic shift far beyond the immediate field.

## The AlphaFold concept

AlphaFold combines numerous deep learning innovations to leverage the combined knowledge from 50 years of experimental science, stored in sequence and structure databases. It relies heavily on multi‐sequence analysis using information on conserved peptide structures, but also on evolutionarily coupled residues. This idea of co‐evolution is relatively simple: If two residues are close in space and interact with each other—even if they are far apart in the amino acid sequence—they will stick together during evolution to preserve structure and function. The AlphaFold team also made several ingenious innovations, creatively using concepts and algorithms originally from natural language processing, and algorithmic choices on using rotationally invariant functions for linking the one‐dimensional sequence to three‐dimensional structure space (Jumper *et al*, 2021; nicely reviewed in: Bouatta *et al*, 2021). With such innovations and massive amounts of processing power and time, the AI was able to learn how an amino acid sequence folds into space.

The wealth of structures now available in the AlphaFold database is a wonderful resource. It includes an interactive graphical representation of the structure with a color‐coding scheme to indicate how confidently the position of each amino acid is predicted. A matrix further indicates the confidence in inter‐residue distances. The presentation makes it easy to explore and download any protein of interest, but also highlights the uncertainties and potential pitfalls of the predicted models.

The AlphaFold database contains near‐perfect predictions for the folded part of many proteins. However, it does not do so well on parts where fewer sequences are available for alignment, and of course on regions that are natively unfolded. It also struggles with protein interface residues, as the partner regions—either in homo‐ or in hetero‐multimers—are not available in the computational folding process. These regions are shown as long loops and are placed randomly with respect to the folded parts, which is reflected in the orange‐red coloring and clearly indicated in the matrix.

**Figure 1:**
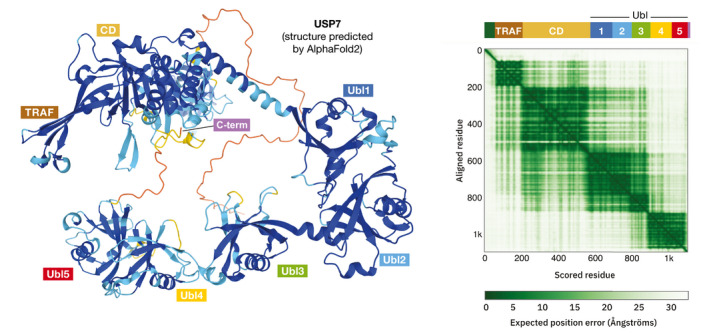
The AlphaFold prediction for USP7 (Left) A ribbon model of USP7 exported directly from the EBI Web server; the coloring ranges from blue (high confidence) to red (low confidence); the domain names are annotated manually. (Right) The AlphaFold matrix showing the expected position error for each residue in the sequence; a detailed explanation of the matrix is available at the EBI Web server; the top bar with domain annotation and names was added manually (figures downloaded from the AlphaFold webserver).

An interesting feature of the AlphaFold models is that they do not only provide incredibly accurate models of individual folding units (domains), but also give indications of dynamic movement between them. A nice example is USP7, a complex enzyme that consists of multiple domains that undergo dynamic conformational changes. Figure 1 shows the AlphaFold prediction with the contact matrix, where individual domains are clearly visible as dark green squares. Interactions between domains also show up: The N‐terminal TRAF domain preferentially interacts with the catalytic (CD) domain. The five ubiquitin‐like domains (UBLs) that follow form two groups, Ubl1‐3 and Ubl4‐5, and it is clear from the structure that the first three interact well with the CD, while the last two do not. Harder to see, but still visible, is the fact that the C‐terminal peptide interacts with the catalytic domain, an important feature for the regulation of activity in this protein. Structural analysis, including small‐angle X‐ray scattering (SAXS), X‐ray crystallography, and NMR spectroscopy of different domain combinations and their interactions, agrees with this prediction, as do previous models of the full‐length protein (Kim *et al*, 2016).

## Current limitations of the models

AlphaFold models have several limitations though. The most obvious one is that interactions with partner proteins or multimers are currently not in the database. However, RoseTTAfold (Baek *et al*, 2021) was already dealing with multimers, and a recent pre‐print presents AlphaFold‐Multimer, able to predict both homomeric and heteromeric interfaces, although still with varying accuracy (preprint: Evans *et al*, 2021) AI also does not predict several other important aspects of protein structures: metal ions, cofactors, and other ligands. Post‐translational modifications, such as glycosylation or phosphorylation, or DNA, RNA, and their complexes, are also absent. In addition, amino acid side chains are not always accurately placed. Each of those features may be crucial for protein function, and many of these are necessary for the integrity of the fold.

**Figure 2:**
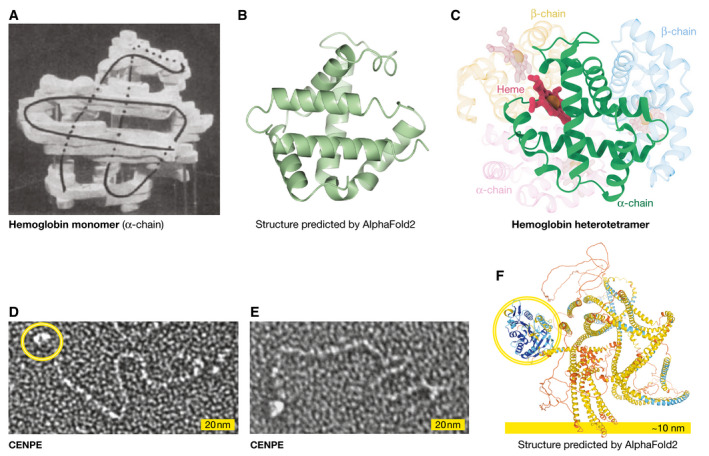
Limitations of AlphaFold models (A) The hemoglobin α‐chain monomer structure by Max Perutz and co‐workers; from Perutz *et al* (1960) Nature 185: 416–22, with permission from Springer Nature; (B) the AlphaFold model, predicted correctly, but lacking ligands and partners, displayed in the same orientation (created by CCP4MG (McNicholas *et al*, 2011); (C) a diagram of the hemoglobin fold that is a heterotetramer of two α and two β chains, each containing a heme coordinating a Fe^2+^ ion. (D,E) an electron micrograph (kindly provided by Y. Kim and D Cleveland) of CENPE showing the motor domain (yellow circle) and the coiled‐coil region that extends several hundreds of Ångström; (F) the AlphaFold model showing the motor domain (yellow circle) that is predicted with high confidence, and the “warped” set of helices that are predicted with low confidence and do not agree with the experimental data (image downloaded from the AlphaFold Web server).

Notably, despite these limitations, AlphaFold does correctly predict the iconic fold of the hemoglobin chain (Fig 2A–C). The lack of either the heme group or its tetramerization partners, all of which are essential for folding, does not stop AlphaFold from perfectly predicting the fold of the α‐chain. AlphaFold has not learned from ligands and is actually not aware of the actual energy minima that are essential for folding in real life. In reality, AlphaFold has not solved the folding problem as it would occur in solution or in a cell, but it has provided a practical solution: It has learned the results of folding at the amino acid residue contact level and can therefore accurately predict a single‐chain hemoglobin fold that would never exist on its own or in the absence of the heme cofactor in nature.

Another educational example is the CENP‐E kinesin model (Fig 2D–F). While the structure of the motor domain is well predicted, the flexible coiled coil, which we know to exist in various elongated forms from electron microscopy data and secondary structure predictions (Vitre *et al*, 2014), appears folded like a ball (thankfully colored orange to show the uncertainty of the prediction), which does not represent a biologically and functionally relevant state. As AlphaFold has predicted on the basis of the monomer, it has not resolved the coiled coil structure, the fold of which depends on dimerization. Should AlphaFold‐Multimer be able to predict coiled‐coil dimers, as it will, it remains to be seen if it will also predict elongated structures that are not represented in the PDB.

It is always good to keep in mind that AI relies, to some extent, on what has been seen before. That is why, for instance, the hemoglobin fold is reproduced well, but elongated structures are typically missed. Novel and unexpected structures may therefore not be predictable.

Many other limitations can be considered “teething problems”. Solutions for completing AI models with essential cofactors and common ligands will appear soon. One would also expect that prediction of DNA/RNA complexes should eventually become feasible. The increasing ability of both RoseTTAfold and AlphaFold‐Multimer in predicting complexes, hold great promise for solving—or at the least creating testable hypotheses—or at the least creating testable hypotheses—for more complicated biological problems for more complicated biological problems.

Even now, AlphaFold models are already a very useful resource. However, it is critical, as always, that users take the limitations of the method into account. If structure predictions are used and interpreted naively, it can lead to erroneous hypotheses or blatantly wrong mechanistic models. It is therefore essential to use the prediction confidence charts as it helps to understand which parts are likely to be real and which should be ignored. This information is not only presented clearly in the Web interface, and it is also embedded in the PDB file in the B‐factor column.

## Beyond AlphaFold

**Figure 3:**
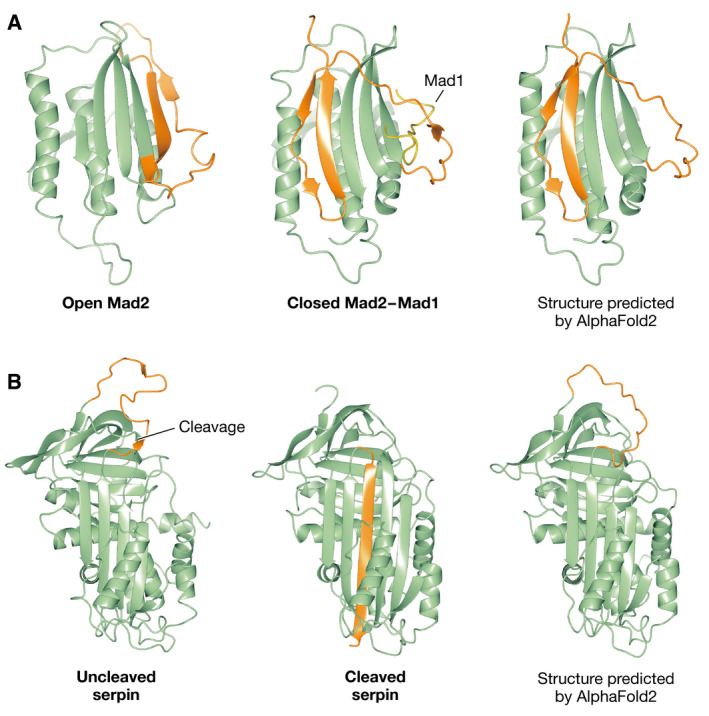
Folding rearrangements (A) The open Mad2 solution structure determined by NMR (left) undergoes a rearrangement of two beta strands (shown in orange), in which Mad2 embraces the Mad1 partner (shown as a yellow ribbon), a structure that was determined by X‐ray crystallography (middle); AlphaFold2 erroneously predicts the structure of the refolded complex with Mad1 as the native structure of Mad2 (right). (B) The structure of an uncleaved Serpin (left) with a long loop (shown in orange), which after proteolytic cleavage inserts as a β‐strand in the middle of the β‐sheet; AlphaFold correctly predicts the structure of the uncleaved protein. All figure panels were created by CCP4MG.

**Figure 4:**
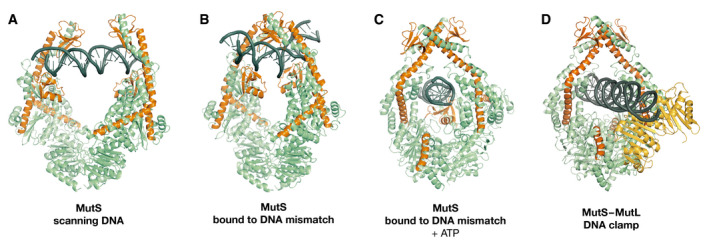
Functional states of MutS Structures of scanning MutS (A), mismatch‐bound MutS (B), transition‐state MutS (C), and MutL^LN40^‐bound MutS (D). Monomer A is shown in green, with monomer B in blue, MutL^LN40^ in orange, and DNA in black.

The most important limitation of AlphaFold predictions is that only a single state is predicted, even if hints for multiple states and dynamic behavior are in the data, like for USP7. It is also hard to tell which state of a protein will be captured by the AI. Figure 3 shows two examples: the mitotic protein Mad2 in which two strands undergo a rearrangement to form a “safety belt” that embraces partner Mad1 (Sironi *et al*, 2002): AlphaFold erroneously captures the complex state for Mad2 alone. The other example is a serine protease inhibitor (Serpin) where a loop inserts as a strand in the middle of a β‐sheet following proteolytic cleavage (Stein *et al*, 1990). AlphaFold correctly captures the uncleaved state. Another case where a protein makes major rearrangements is shown in Fig 4: Different states of the DNA mismatch protein MutS that have been determined from a series of cryo‐EM experiments (Fernandez‐Leiro *et al*, 2021). AI only predicts a single state and does not explain the functional behavior.

There are also protein regions that AlphaFold cannot predict, and it will be important to find out why. Which regions will have stable folds, in isolation or in complexes, that AlphaFold has missed? Which fraction will consist of truly intrinsically disordered regions (IDRs) that are used for instance in phase separation? As more structures and sequences become available, and as the methods further improve, it seems likely that the fraction of poorly predicted protein will decrease. A major step forward will come from inclusion of multimers and complexes in the predictions, but there may be other regions that adopt defined states only temporarily under very specific conditions.

Yet, AlphaFold models are already useful for many practical aspects of structural biology: to design better protein expression experiments (https://ccd.rhpc.nki.nl/); to solve experimental structures faster, especially for X‐ray crystallography (Millán *et al*, 2021); to overcome tedious model building steps in experimental crystallographic and cryo‐EM maps; and to interpret lower‐resolution cryo‐EM maps, where structure solution is limited by dynamic variability.

Above all, the analysis of the models themselves can generate new and testable hypotheses about protein function. This actually is the great joy of structural biology: to derive mechanistic insight from a protein structure. Here, we have a whole candy store of new structures to play with. There is a lot to do, and many of us are already busy designing mutants and experiments to test new ideas and hypotheses based on the AlphaFold models themselves. In many cases the biochemical, biophysical or cellular validation, will be enough and further structural validation may not be required.

In this sense, AI provides structural biologists with a new technique, bringing the fun of structure‐gazing without the effort of experimental work. However, as models now become readily available, some of the joy in the discovery of a new structure will be gone, and so might be the drive and enthusiasm to interpret it. Moreover, there is a danger that people will be less inclined to prepare high‐quality protein, which is actually useful for many more experiments than determining structures. It will thus be crucial to educate the next generation of biologists to learn how to critically analyze the predicted fold, notice new interactions, and to get to know each protein of interest in sufficient detail, so as to differentiate between “bugs” and “features”.

## Impact and opportunities for drug discovery

High‐resolution protein structures are extremely useful for drug discovery. Current drug development uses structures at every step of the process: from initiating discovery by fragment screening in crystals or in solution, to improving ligand interactions, to engineering high‐affinity ligands with favorable drug‐like properties. The heavy investment of the pharmaceutical industry into X‐ray crystallography and NMR spectroscopy in the past, and in cryo‐EM nowadays, is testament to the importance of structures. Nonetheless, it would be obviously attractive if the experimental process could be entirely replaced by a computational approach. The current AlphaFold implementation does not yet have the accuracy that is necessary for drug discovery. More importantly, the ability to accurately predict novel ligand interactions may be limited, as publicly available data for binding small molecules are scarce. This reduces the opportunities for deep learning. More data are potentially available in private databanks, and the expected acceleration in fragment screening by X‐ray crystallography and cryo‐EM could change this situation.

For now, drug discovery will require experimental analysis, and here, the AI revolution may actually be counterproductive. The easy availability of computational models might result in fewer target proteins for which good purification protocols are developed, and fewer well‐diffracting crystals or well‐developed sample preparation processes for cryo‐EM. All these are necessary for drug discovery as X‐ray crystallography remains the method of choice to study structures in complex with small molecules, while cryo‐EM becomes the method of choice for studying complexes with biological therapeutics, such as antibodies or nanobodies.

## An example for an AI‐fueled paradigm‐shift in science

The AI solution to the protein folding problem is not only one of the most important advances that happened to structural biology, and it is arguably the most ground‐breaking application of AI in science to date. As such, we can expect that many of its methodological ideas will find their ways into AI applications beyond structural biology. However, with all revolutions peril lurks along the way. It is important to keep in mind, that it does not matter how correct AI thinks a model is, “if it doesn't agree with experiments, it's wrong”, to paraphrase Richard Feynman. For this reason alone, experiments remain essential. Similarly, pathologists and oncologists should remain critical of AI analyses of biopsies or scans, evaluate the results, and communicate them to patients. It is likely that the next generation of structural biologists will no longer be mainly experts in experimental methods, but will primarily interpret, design, and perform experiments based on structures, to prove or disprove mechanisms in biology or to design new protein functions or therapeutics.

Experimental structure determination will not become obsolete for the foreseeable decades. The more intricate questions about conformational states, dynamics, and transient interactions will still need attention and experiments to answer. It is thus important that funders—and peer‐reviewers—do not come to believe that “the folding problem has been solved”. They should continue to invest both in the critical infrastructure needed for experimental structural biology and the research that uses experimental structure determination, where needed.

The protein folding example will provide valuable lessons in how we can benefit from the application of AI models without creating an irrevocable dependence on them

As AI applications become more mainstream in modern society, from AI‐based chat‐bots, virtual assistants, self‐driving cars or “intelligent” robotic vacuum cleaners and lawn mowers, the problem of transforming the roles of the people who had done these tasks before will be ever present. The protein folding example will provide valuable lessons in how we can benefit from the application of AI models without creating an irrevocable dependence on them. At the end of the day, the development of deep learning algorithms is no different from human learning: We all learn from experiments, every day.
